# Cone-beam computed tomography for primary investigation of wrist trauma provides a new map of fractures of carpal bones

**DOI:** 10.1177/17531934211001730

**Published:** 2021-03-24

**Authors:** Mamoun Krayem, Claudia Weber Lensing, Lotta Fornander

**Affiliations:** 1 Department of Radiology, Norrköping, Sweden; 2 Department of Health, Medicine and Caring Sciences, Linköping University, Norrköping, Sweden; 3 Department of Orthopedic Surgery, Norrköping, Sweden; 4 Department of Biomedical and Clinical Sciences, Linköping University, Norrköping, Sweden

**Keywords:** Carpal bone, scaphoid, fracture, cone-beam computed tomography, radiography

## Abstract

In 2016, our primary modality for radiological examination of wrist trauma, was changed from radiography to cone-beam computed tomography (CBCT). This is a retrospective survey of carpal bone fractures detected by CBCT during 6 months in 2016/2017, compared with those found on conventional radiographs during 6 months in 2013/2014. The incidence of carpal fractures was three times higher during the CBCT period (92/100,000 per year) compared with the radiography period (29/100,000 per year) and the spectrum of anatomical locations was different between the two periods, with fractures of the lunate (*n* = 6), trapezium (*n* = 9), trapezoid (*n* = 4) and capitate (*n* = 1) detected by CBCT, in contrast to no fractures of these bones diagnosed during the 6 months radiography period. We suggest a more liberal use of CBCT for examination of wrist trauma considering the benefits of being able to give patients a correct primary diagnosis, treatment and prognosis.

**Level of evidence:** III

## Introduction

Patients presenting with wrist trauma are routinely examined using conventional radiography. Plain radiography reveals approximately 75% of distal radius fractures (Balci et al., 2015) and 70% or less, of scaphoid fractures (Balci et al., 2015; Blum et al., 2007; Jørgsholm et al., 2013). Fractures of other carpal bones are also often not diagnosed, but have gained less interest in the literature and in clinical practice, although most of these fractures require non-surgical or surgical treatment to avoid morbidity and disabling sequelae (Pan et al., 2016).

Multidetector computed tomography (MDCT) has proven superior to conventional radiography in fracture detection but the high radiation dose limits its routine use (De Smet et al., 2015). Cone-beam computed tomography (CBCT) is an imaging technique generating a higher spatial resolution at a lower radiation dose than MDCT (De Smet et al., 2015) and has shown to be more accurate than conventional radiography for the diagnosis of fractures of the carpal bones (De Smet et al., 2015; Gibney et al., 2019; Neubauer et al., 2018). The effective radiation dose of CBCT is only 0.7–2.4 times higher than conventional radiography (Koivisto et al., 2018). Scanning is faster to perform than radiography with specific wrist projections (Huang et al., 2015). Reporting takes approximately as long as for a MDCT, depending on the experience of the radiologist. The CBCT equipment does not require much space and is therefore suitable for an emergency department environment.

In October 2016, our hospital’s routine for radiological examination of wrist trauma was changed from conventional radiography to CBCT. Since then, all patients above the age of 14 years with wrist trauma have been evaluated with CBCT, without prior radiography. An average of 1086 CBCT wrist examinations are performed per year (2017–2020), which gives us the benefit of having access to a large number of CBCT wrist studies for evaluation.

The purpose of this study was to calculate the incidence of carpal bone fractures and to describe the fractures found during a period of time when CBCT was used as the primary investigation for wrist trauma and to compare that with the incidence and characteristics of carpal bone fractures in a time period when conventional radiography was used.

## Methods

The study was carried out at Vrinnevi Hospital, Norrköping, Sweden, which serves a total population of 170,000 inhabitants, in the southeast of Sweden. It was approved by the national Swedish ethics committee (Registration number 2020-00703).

### Patients

The study is a retrospective survey of carpal bone fractures assessed by CBCT, during a time period of 6 months in 2016/2017 and the results are compared with the results of conventional wrist radiographs obtained during the same time period in 2013/2014. The periods 1 December 2016 to 28 February 2017 (winter) and 1 June 2017 to 31 August 2017 (summer) were chosen for evaluation of the results of the CBCT examinations. For comparison, the corresponding winter period of 2013/2014 (1 December 2013 to 2 February 2014) and the summer period of 2014 (1 June 2014 to 31 August 2014) were chosen for evaluation of conventional radiography of the wrist.

All cases assessed during these time periods were eligible for inclusion. Six-hundred and three cases of wrist trauma assessed by CBCT and 944 cases assessed by conventional radiography were found. After exclusions according to the criteria listed below, 415 CBCT cases and 643 conventional radiography cases were included and assessed. Exclusion criteria were: (1) age ≤14 years; (2) non-traumatic cases, for example osteoarthritis, osteomyelitis, tumours; (3) follow-up of previous trauma cases; (4) examinations performed for preoperative planning; (5) examinations with artefacts, accompanying severe arthrosis or calcification, impairing interpretation; (6) cases with remaining uncertainty regarding diagnosis after final reviewing of the images (three cases excluded).

The difference in number of cases included between the two modalities is presumably related to the current routine of continued use of conventional radiography in patients investigated for simultaneous injuries in other parts of the body, for logistic reasons.

### Radiography

During the winter and summer period of 2013–2014, all patients underwent plain radiography including posteroanterior (PA) and lateral views of the wrist in the neutral position in addition to four dedicated projections with special tube angulation when a scaphoid fracture was suspected. The digital images were obtained with a flat panel detector, with voltage of 55 kV, tube current of 3 mA and a 115-cm film-to-focus distance. The estimated effective radiation dose for a plain wrist radiograph was 9.5 mGy for two views and 24.4 mGy for all six views.

### CBCT

CBCT images were obtained using a CBCT scanner (Planmed Verity, Helsinki, Finland) designed for extremity scanning purposes. The examination was carried out with the patient in a seated position with the hand placed in the scanner aperture and stabilized by supporting pillows. The examination was conducted using a voltage of 90 kV and a current of 6 mA. The CBCT scans were reconstructed to a slice thickness of 0.2 mm with the field of view set to 15 × 15 cm. The total scan acquisition time was approximately 30 s. Three orthogonal planes (axial, coronal and sagittal) were reconstructed and saved. The radiation dose (DLP) for a CBCT scan was 35 mGy.

### Image analysis

According to clinical routine, images were originally reported by two radiologists. For the scope of this article, all cases were then reviewed by a radiologist with 20 years’ experience and findings of fractures of the radius, ulna, metacarpal and carpal bones were documented. If discordance between the original opinion and the opinion of the reviewer arose, a third opinion of another similarly experienced radiologist was requested.

Further information was recorded in all cases with carpal bone fractures, including the bone fractured, gender, age, season, mechanism of injury (as stated in the radiology request), type of fracture (full thickness (defined as a fracture involving two opposing cortices and passing through the full thickness of the bone) or avulsion), displacement, and the relation of carpal fractures to each other, and to radius/ulna and metacarpal fractures.

### Statistical methods

Incidence rates for occurrence of carpal fractures were calculated, based on the population of 170,000 inhabitants served by our hospital. Confidence intervals (95%) were calculated for the incidence rates of the two time periods.

## Results

### Incidence

Seventy-eight carpal bone fractures were found using CBCT in the 6 months study period of 2016/2017 (1 December 2016 to 28 February 2017 and 1 June 2017 to 31 August 2017). The incidence of carpal bone fractures was calculated to be 92/100,000 per year (78–107, 95% CI). In the 6 months study period of 2013/2014 (1 December 2013 to 2 February 2014 and 1 June 2014 to 31 August 2014) radiography revealed 25 carpal bone fractures, which corresponds to an incidence of 29/100,000 per year (22–39, 95% CI).

### Demographics

**Figure 1.**
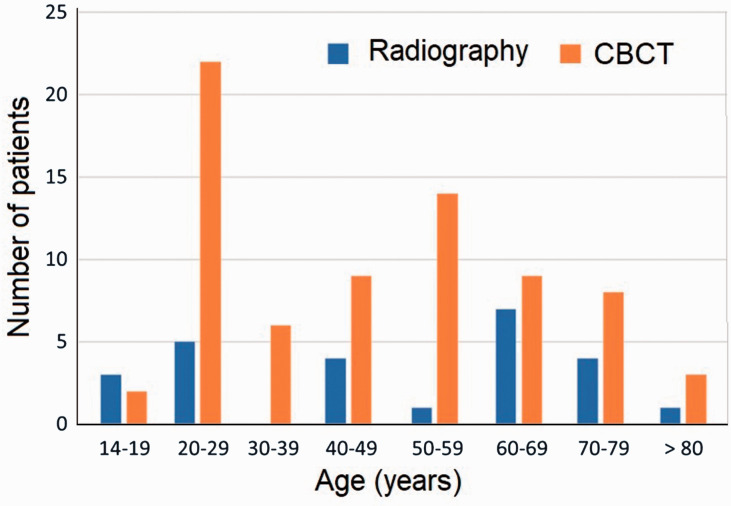
Age distribution of patients with fractures of carpal bones diagnosed by conventional radiography (blue) and CBCT (orange).

Mean age was 46 years (range 14–89 years, SD 19.8) in the group examined by CBCT and 49 years (range 14–82 years, SD 21.6) in the radiography group. The difference was not significant. However, the distribution of carpal bone fractures between age groups differed between the two modalities (Figure 1). There was a gender preponderance of carpal fractures towards males. Using CBCT the male/female ratio was calculated to 1.7:1, while using radiography the ratio was 2.1:1.

### Mechanism of injury

In 80% of cases with carpal fracture, a fall from standing height (i.e. low energy trauma) was the cause of injury. Insufficient information about the landing was available to analyse the position in which the wrist was hit. In eight cases the mechanism of trauma was stated as unclear or not provided at all and in only a few cases was the injury caused by high-energy trauma, such as road traffic accidents, bicycle accidents or acts of aggression.

### Anatomical distribution of carpal fractures

**Figure 2.**
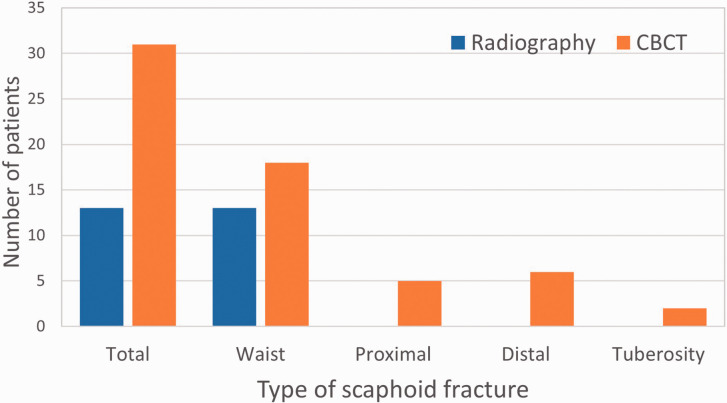
Total number and distribution of location of scaphoid fractures detected by conventional radiography (blue) and CBCT (orange).

The scaphoid was the most frequently fractured carpal bone both in the CBCT and the radiography groups. Analysis of cases with scaphoid fractures in the CBCT group revealed 18 scaphoid waist fractures, six distal, five proximal and two tuberosity fractures (Figure 2). Most cases occurred in men (20 out of 31 cases) in the 2nd and 3rd decades of life. In contrast, all cases in the radiography group were fractures of the waist of the scaphoid (Figure 2).

**Figure 3.**
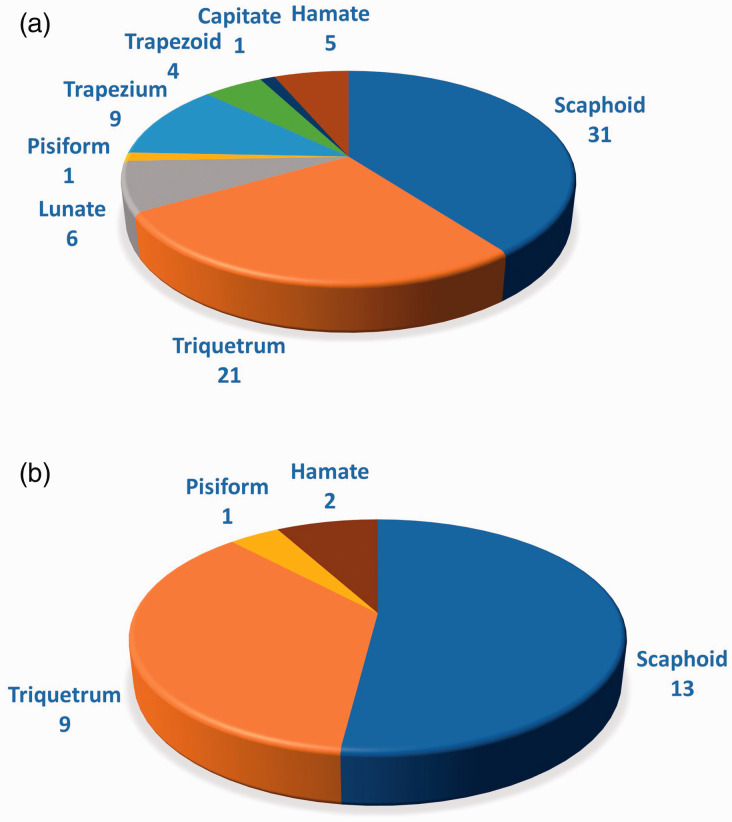
Anatomic distribution of fractures of carpal bones identified using (a) CBCT and (b) conventional radiography.

The second most fractured carpal bone in both groups was the triquetrum. In the CBCT group we found 21 triquetral fractures: 16 dorsal, four body and one volar fracture. No significant sex predilection was noted (12 men and nine women). The fracture was found in all age groups with a slight predilection to old age. The distribution of fractures between the remaining six carpal bones diverged between the two modalities (Figure 3).

In three cases examined with CBCT, two or more concomitant fractures of other carpal bones were found. All of these included a scaphoid fracture. No concomitant fractures were detected by radiography. Among the 78 cases of carpal bone fractures diagnosed with CBCT, we found eight cases with concomitant radius fractures and three cases with concomitant metacarpal base fractures. In contrast, with radiography, there was only one case with a concomitant radius fracture and one case with a concomitant metacarpal fracture among the 25 carpal fractures.

### Type of fracture

During the CBCT examination period of 2016/2017, 47 of the carpal fractures were full thickness fractures, compared with 14 during the radiography examination period of 2013/2014. Furthermore 27 fractures diagnosed with CBCT were displaced fractures compared with 13 fractures detected by radiography.

## Discussion

**Figure 4.**
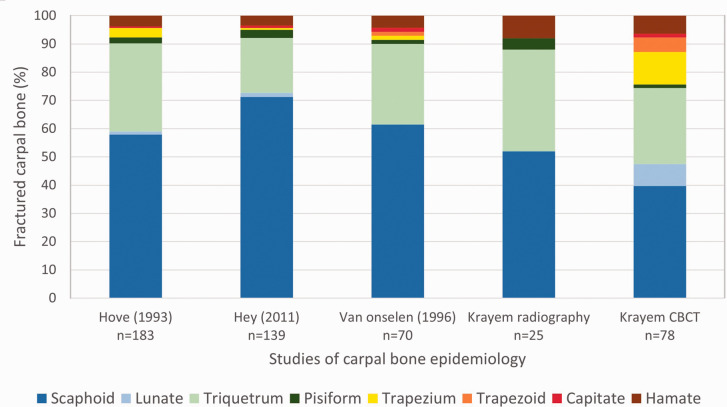
Distribution of carpal bones fractured. Comparison of the results of our study (Krayem) to three previous radiography studies (Hey et al., 2011; Hove, 1993; van Onselen et al., 2003) of epidemiology of fractures of carpal bones.

By using CBCT for primary examination of wrist trauma, we found an incidence of fractures of carpal bones three times the incidence found during the radiography period. CBCT detected fractures in all carpal bones, whereas with radiography, only fractures of the scaphoid, triquetrum, pisiform and hamate were found. Our study consequently presents a different spectrum of carpal fractures compared with that reported in previous radiography studies (Figure 4).

Fractures of the trapezium, trapezoid and the lunate seem to be far more common than previously recognized. Gibney et al. (2019) stated, in their CBCT study of patients with wrist pain and normal radiographs, that the trapezium is the most common radiographically occult radiocarpal fracture. Lunate fractures are described in the literature as rare, comprising 0.5–1% of all carpal fractures and when they are found, they are usually part of a more complex fracture pattern with concomitant fractures, of other carpal bones or the distal radius, and ligament injuries leading to midcarpal instability (Shunmugam et al., 2018). Our study adds information on the existence of isolated lunate fractures, which comprised 8% of the carpal fractures found during the CBCT period. Lunate fractures without joint subluxation can often be treated with a plaster cast, but require radiological follow-up, because of the risk for non-union (see scaphoid fractures) (Pan et al., 2016).

The usage of CT for wrist examination has been questioned, some arguing that it will merely add information about small avulsions of dubious significance. However, this study shows that CBCT reveals full-thickness fractures to a greater extent than radiography. The differences in number of full-thickness fractures between CBCT and radiography could also be related to the poorer sensitivity of radiography to detect fractures of carpal bones beyond fractures of the scaphoid and triquetrum. This increases the impact of triquetral dorsal flake fractures, easily visualized by radiography, on the ratio between avulsion fractures and full-thickness fractures in the radiography group. The higher proportion of displaced fractures using radiography can be attributed to the lack of sensitivity of radiography for detecting non-displaced fractures. In summary, the additional fractures found with CBCT were often full-thickness fractures requiring treatment with a plaster cast and sometimes surgery.

We found more than twice the number of scaphoid fractures with CBCT (31) compared with radiography (13). Similar numbers of scaphoid waist fractures were found in both groups, but CBCT revealed additional fractures of the distal and proximal pole. In a previous (radiography) study, 64% of scaphoid fractures occurred at the waist of the scaphoid, 31% at the distal pole (of which 58% were tubercle avulsions) and only 4.6% involved the proximal pole (Garala et al., 2016). We found five proximal pole fractures by CBCT, which represents 16% of the scaphoid fractures, hence a high proportion in comparison with both our own radiography results and compared with the results of Garala et al. (2016). We suggest that the proportion of proximal pole fractures of the scaphoid is underestimated when using radiography for diagnosis and that they are at risk of neglect if radiography is not supplemented with other radiological modalities. Fractures of the proximal pole have a high risk of non-union due to both the disrupted retrograde blood supply and to instability and hence surgical treatment is often advocated (Clementson et al., 2020).

A limitation of this study is its retrospective design using two different time periods for comparison of the incidence of carpal fractures. A prospective study examining the same patients with both radiography and CBCT would possibly give more accurate and comparable incidence rates. The number of patients included in this study is, however, quite large and a prospective study with the same sample size would be difficult to implement, costly and potentially ethically questionable because of the increase in radiation dose to the patients included.

In conclusion, our study provides new knowledge on the incidence of carpal bone fractures by using CBCT for primary investigation of wrist trauma. Fractures of carpal bones appear to be more common than previously reported and the distribution of anatomical location differs considerably compared with when radiography was used. Based on these results we advocate more liberal use of CBCT for examination of wrist trauma, considering the benefits of being able to give patients a correct primary diagnosis, treatment and prognosis at the cost of a negligible increase in radiation dose compared with conventional radiography.
